# Analysis of superfamily specific profile-profile recognition accuracy

**DOI:** 10.1186/1471-2105-5-200

**Published:** 2004-12-16

**Authors:** James A Casbon, Mansoor AS Saqi

**Affiliations:** 1 Bioinformatics Group, Centre for Infectious Disease, Institute of Cell and Molecular Science, Queen Mary's School of Medicine and Dentistry, University of London, 32 Newark St, London E1 2AA, UK

## Abstract

**Background:**

Annotation of sequences that share little similarity to sequences of known function remains a major obstacle in genome annotation. Some of the best methods of detecting remote relationships between protein sequences are based on matching sequence profiles. We analyse the superfamily specific performance of sequence profile-profile matching. Our benchmark consists of a set of 16 protein superfamilies that are highly diverse at the sequence level. We relate the performance to the number of sequences in the profiles, the profile diversity and the extent of structural conservation in the superfamily.

**Results:**

The performance varies greatly between superfamilies with the truncated receiver operating characteristic, *ROC*_10_, varying from 0.95 down to 0.01. These large differences persist even when the profiles are trimmed to approximately the same level of diversity.

**Conclusions:**

Although the number of sequences in the profile (profile width) and degree of sequence variation within positions in the profile (profile diversity) contribute to accurate detection there are other superfamily specific factors.

## Background

Currently some of the best methods for detecting relationships between protein sequences below the so-called twilight zone of sequence similarity are offered by iterative search algorithms such as PSI-BLAST [1] which, in effect, compare sequences to a profile. More recently profile-profile matching protocols [2-5] have been shown to offer considerable benefits over sequence-profile matching.

Here, we examine how the performance of remote homolog detection by profile-profile methods varies between particular superfamilies. Since superfamilies are believed to constitute sets of remote homologs, detection of same-superfamily relationships is an important task for bioinformatics, and with the increasing number of structures becoming available, improvement in this area will help build a complete structural map of sequence space. In this paper, we use a set of superfamilies that are very sequence diverse to benchmark profile-profile methods. By sequence diverse, we mean that the superfamily has many domains that show no detectable sequence similarity to each other; this lack of detectable sequence similarity means this set is a difficult benchmark for remote homolog detection methods.

Previous work has shown that the performance of profile-profile methods is chiefly determined by the width and diversity of the profiles. By *profile width*, we mean the number of sequences in the profile, defined in contrast to profile length and by diversity we mean the degree of sequence variation within positions in the profile. In particular, Panchenko suggested that there may be an optimum level of profile diversity [6], whilst Grishin suggested that the inclusion of as many related sequences as possible gives maximum performance [7].

We examine the performance of profile-profile matching with regard to specific superfamilies with both the full profiles generated from a PSI-BLAST search, and with profiles that are trimmed to similar width and diversity. Significant differences in recognition performance exist between superfamilies for both the full and trimmed profiles. This suggests that performance of profile-profile matching is not simply a function of profile width and diversity. We examine how the performance relates to the structural diversity of superfamilies and find that structurally conserved superfamilies are recognised more successfully than structurally diverse superfamilies.

## Results

### Width and diversity of profiles

Table 1 shows the width and diversity for the full and trimmed profiles. The table shows average profile width in for each superfamily in the dataset before and after trimming (as detailed in the Methods section). The table also shows average Neff (defined as the total number of different amino acids in a given column of a profile [1,6,7]) across all non-gapped columns for each profile in the superfamily. The full profiles show considerable variation in both size and diversity of the profiles. The trimmed profiles, however, are much more similar in both width and diversity, with values of Neff consistently around three.

### Superfamily specific performance of remote homolog detection

**Figure 1:**
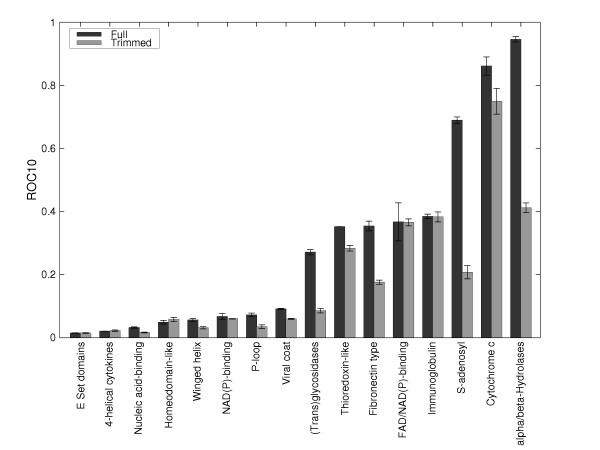
*ROC*_10 _values for each superfamily in the dataset for full and trimmed profiles.

Figure 1 shows the value of the performance measure *ROC*_10 _(see Methods for definition) for each superfamily. The figure shows that there is a large variation in performance with respect to superfamily for both the full profiles and the trimmed profiles.

For the full profiles, the alpha/beta-Hydrolases, Cytochrome c and S-adenosyl superfamilies perform well, all having with *ROC*_10 _values ≥ 0.7, the fibronectin, thioredoxin-like, (trans)glycosidases, immunoglobulin and FAD/NAD(P)-binding have *ROC*_10 _> 0.2 and the remaining 8 superfamilies all perform poorly, having a performance less than 0.1.

After trimming, although performance is reduced, the overall pattern of performance still remains. All the well recognised superfamilies (with the exception of the (trans)glycosidases and thioredoxin-like) still show *ROC*_10 _values greater than 0.2, while the rest are still less than 0.1.

The fact that the performance varies greatly between superfamilies despite the trimming of the profiles indicates that the profile generation is not the only limiting step in the performance of profile-profile methods. One might have thought that, for instance, the bad recognition of 4-helical cytokines is due to the small number of homologs drawn from the profile-building stage. Whilst this still may be true, it is not necessarily true: the Cytochrome c superfamily still shows a *ROC*_10 _of 0.7 when using trimmed profiles despite having, on average, less than 20 sequences in the profile.

### Structural diversity

**Figure 2:**
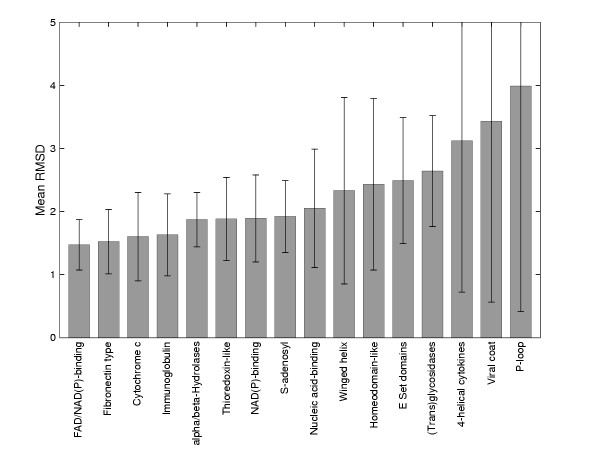
Mean RMSD values for superfamilies in the dataset. Error bars show one standard deviation.

Figure 2 shows the average root mean square deviation (RMSD) across each superfamily in our dataset. As can be seen, there is a large range in the degree of structural diversity across the dataset: some superfamilies are highly structurally conserved showing a narrow range of small RMSDs whilst other show large mean RMSDs with large deviations from the mean. For example, the FAD-NAD(P)-binding SCOP super-family contains 21 domains in the astral_10 data set, and despite the low sequence identity there is high structural conservation with an average RMSd of 1.47Å. Furthermore, the range of RMSDs within this super-family is very small, generally within 0.5-2Å. By comparison, super-families such as the P-loop containing nucleotide triphosphate hydrolases, the (Trans)Glycosidases and the Viral-coat and capsid proteins are very structurally diverse, having high average RMSds with the distribution of RMSds generally higher than 1.5Å, and with a long tail.

### Relation between structural diversity, sequence conservation and recognition performance

**Figure 3:**
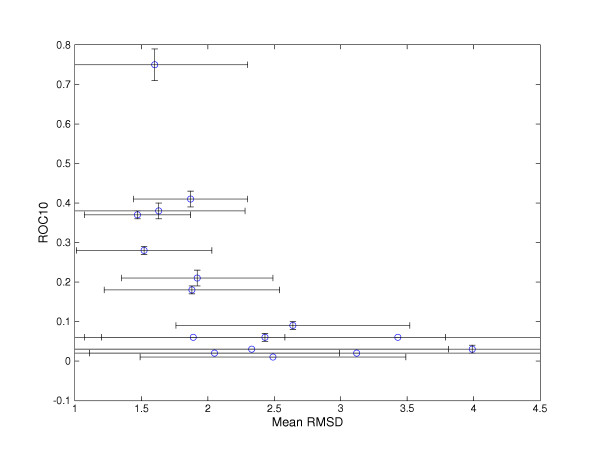
*ROC*_10 _of the trimmed profiles versus average pairwise RMSD. Error bars show one standard deviation.

Figure 3 shows a scatter of mean RMSD against *ROC*_10 _for each superfamily. The figure shows a correlation between the mean RMSD of each super-family and its *ROC*_10 _value. The figure shows that superfamilies with a mean RMSD of less than 2 Å tend to be well recognised by profile-profile methods, whilst the structurally diverse superfamilies are not.

It may be the case that despite the absence of any discernible global sequence similarity within our dataset some local patterns of conservation do exist. These patterns may be present more strongly in some superfamilies than in others. In order to examine this possibility we constructed multiple structure based sequence alignments for each of the 16 superfamilies and then looked down the columns of the multiple sequence alignments to examine the extent of conservation at each position (see Methods section).

**Figure 4:**
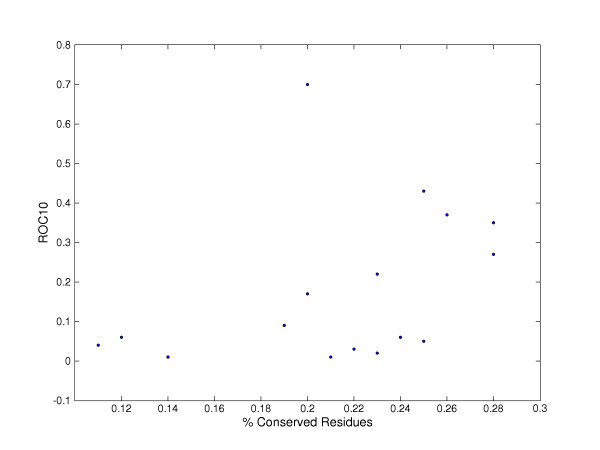
*ROC*_10 _of the trimmed profiles versus conservation for superfamilies in our dataset.

Figure 4 shows a plot of performance (*ROC*_10_) versus conservation. Apart from the cytochrome c superfamily (an outlier with a high *ROC*_10 _of 0.7 despite a conservation score of 0.2 because the superfamily has a conserved CxxCH motif that facilitates detection), the well performing superfamilies (the alpha beta hydrolases, immunoglobulins, FAD/NAD(P)-binding and fibronectin with *ROC*_10 _values for the trimmed profiles of at least 0.25) have conservation measures of greater than 0.25. This suggests that some superfamilies although highly sequence diverse, may retain some patterns of conservation that facilitate recognition. Further investigation of the functional implications of this variation would be a next step.

**Figure 5:**
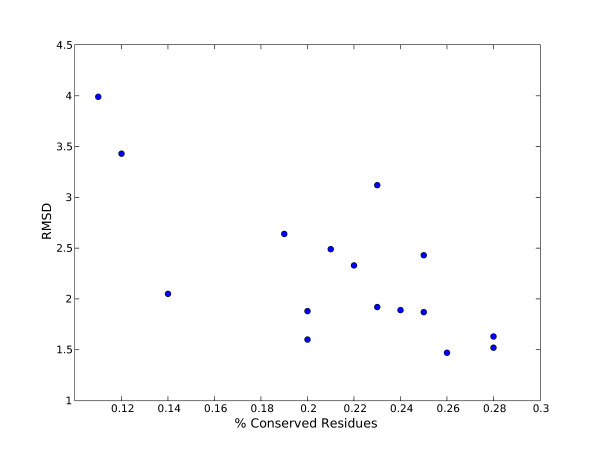
Mean RMSD of the trimmed profiles versus conservation for superfamilies in our dataset.

Figure 5 shows a plot of mean RMSD versus performance (*ROC*_10_). The P-loop and Viral coat superfamilies have low conservation scores and and large structural diversity reflected by high RMSD values. In contrast, the fibronectin and immunoglobulin superfamilies have higher conservation values (both around 0.28) and lower RMSDs (around 1.5Å). However the figure does not show any clear correlation between conservation and RMSD.

## Discussion

Our results suggest that profile profile methods can detect remotely related sequences for some superfamilies significantly better than for others. In our dataset the sequence identity between domains in all the superfamilies is low (not greater than 10% as defined by the ASTRAL). Although the mean width and diversity of the profiles varies across the superfamilies this does not appear to be the only factor contributing to the differences in detection.

The effect of the trimming varied depending on superfamily. For the best performing profile (alpha/beta hydrolases) the trimming reduced the performance by about 50% (from 0.95 to 0.43) but the effect on the rank was small dropping from first place to second. Similarly the trimming impacted significantly on the performance of the S-adenosyl methyl transferases with *ROC*_10 _dropping from 0.70 to 0.22. However trimming had no effect on performance for the FAD/NAD(P)-binding superfamily, and only resulted in a small reduction in performance for the immunoglobulins and the cytochrome c superfamilies. Importantly the membership of the top ranking superfamilies in terms of performance did not change after trimming.

Although the overall level of sequence similarity within our dataset is low (not more than 10% identity) the different superfamilies exhibit different levels of conservation at positions within the multiple structure based alignments. These conserved positions may facilitate recognition. The extent to which they constrain the structures leading to less diverse alignments is unclear. We recognise also that our measure of conservation and also the use of RMSD as a measure of structural diversity both have their shortcomings. It would be interesting to identify and extract a conserved core and represent structural profiles as combination of core profiles separated by regions of variable length.

## Conclusions

There exist large superfamily specific differences in the performance of profile profile matching for the detection of remote sequence relationships. Some superfamilies can be detected far more successfully than others. The width and diversity of the profiles are important factors in successful recognition. However these are not the only factors that contribute to these superfamily specific differences.

## Methods

### Dataset

We took release 1.63 of ASTRAL [8] which provides a filtered version of the SCOP database [9] where no two sequences have a pairwise sequence identity of over 10%. From this, we chose the sequence diverse superfamilies by selecting all superfamilies with more than 20 domains. This resulted in a dataset of 543 domains which only show a random (not greater than 10%) level of sequence similarity. The particular superfamilies used and a summary of their properties is shown in Table 2. Superfamily is a readable description of the superfamily, sunid is the SCOP unique identifier, families is the number of families in superfamily, domains is the number of domains in superfamily, length shows the average length of the domains in the superfamily and RMSD shows average RMSD between members of superfamily.

### Profile generation

For each domain of each of the 16 superfamilies we executed a five round PSI-BLAST [1] run against the protein non redundant protein database nr (dated 5/2/04). We used the "-m6" option to output a multiple alignment and the "e 0.05" to only include hits with e-values less than 0.05 in the alignment. Positions in the multiple alignment that correspond to gaps in the query are removed. We use the resulting multiple alignment as the profile for the query domain.

To produce trimmed profiles, we take the full profile and remove the bottom sequence (corresponding to the most remote homolog) until a stopping criterion is reached. The stopping criterion is based on Neff, a statistic previously used for this task [1,6,7]. Neff is defined as the total number of different amino acids in a given column of a profile. Our stopping criterion was that Neff must be less than 8 in all non-gapped positions in profile, where non-gapped positions are defined as those with a gap content of less than half.

### Profile-profile matching

We use the program COMPASS [2] to perform the profile profile matching. COMPASS performs a local alignment of a query profile to each member of a database of profiles. COMPASS uses a generalisation of PSI-BLAST profile-sequence scoring to score similarities between profiles and estimate the statistical significance of the score of the local alignment.

### Assessing performance

To assess the performance of profile-profile matching, each domain of each of the 16 superfamilies was used as a query and its sequence profile was matched against a library of sequence profiles representing the dataset. A profile database was then created using the 543 profiles. When matching the profile of domain *i *of superfamily *j*, (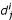), the sequence profile corresponding to 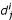 was not included in the sequence profile library. This procedure was carried out twice: firstly with the full profiles, and the again with the trimmed profiles.

We use *ROC*_10 _as a statistic that describes the performance of the profiles for a particular super-family. *ROC*_*n *_is defined as 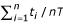, where T is the total number of true hits possible and *t*_*i *_is the number of true positives with a score better than the *ith *false hit. Variance in the *ROC*_10 _statistic was calculated using the method given in [10].

### Structural diversity of superfamilies

To evaluate the structural diversity within each superfamily, each member of a superfamily was structurally compared to every other member. For all the domains in a superfamily we perform pairwise structural alignments using the program SAP [11] to all other domains. Since these domains do not share more than about 10% sequence identity, we would expect that they effectively capture the extent of structural variation within the superfamily. We obtain an average measure of structural similarity (root mean square deviation, RMSD) for each of the 16 superfamilies.

### Structure based multiple alignments

To create a structure based multiple alignment of a superfamily, we first made all pairwise structural comparisons between all pairs within a superfamily using SAP [11,12]. We then created a T-Coffee [13] library for each pairwise comparison, where the score between two equivalenced residues is *i *and *j *at positions *x*_*i*_, *x*_*j *_in the superposition, is defined to be ((1 + *RMSD*)(1 + |*x*_*i *_- *x*_*j*_|))^-1^. A detailed explanation and analysis of this method is given in [14].

### Conservation measure

We used the Taylor Venn diagram [15] to assign residues in a column of the multiple alignment to a given set. The sets are overlapping and they group together amino acids at differing levels of detail (eg the hydrophobic set includes aromatic [FYWH] as a subset). However, we adopted a fairly general measure of conservation and marked a position (column) as conserved if 80% of the residues at that position could be assigned to any one set. The conservation measure for a superfamily was the number of conserved positions divided by the average length of domains in our dataset belonging to that superfamily. Only those columns that contained at least 80% of positions ungapped were considered.

## Authors' contributions

JAC carried out the benchmarking and wrote the necessary code and helped to prepare the manuscript. MASS conceived of the study, provided input into the design and refined the manuscript. All authors read and approved the final manuscript.

## Figures and Tables

**Table 1:** Profile width and Neff for dataset

	Profile Width		Neff	
Superfamily	Full	Trimmed	Full	Trimmed
(Trans)glycosidases	410.4	23.93	13.11	3.21
4-helical cytokines	85.71	43.57	4.3	2.86
alpha/beta-Hydrolases	509.43	22.32	16.32	3.65
Cytochrome c	413.62	18.86	12.64	3.7
E Set domains	182.73	33.27	7.99	3.16
FAD/NAD(P)-binding	616.52	20.57	15.33	3.68
Fibronectin type	1661.67	24.83	11.44	3.55
Homeodomain-like	255.21	39.33	7	3.34
Immunoglobulin	1614.7	69.04	11.33	3.65
NAD(P)-binding	463.14	29.55	12.32	3.27
Nucleic acid-binding	224.09	23.57	8.21	3.11
P-loop	483.03	26.44	11.64	2.92
S-adenosyl	472.42	22.08	14.88	3.22
Thioredoxin-like	471.72	25.28	12.61	3.58
Viral coat	265.28	35.93	6.11	2.96
Winged helix	206.94	24.81	8.11	3.13

**Table 2:** Properties of the dataset

Superfamily	sunid	Families	Domains	Length	RMSD
(Trans)glycosidases	51445	9	30	385.2	2.64
4-helical cytokines	47266	3	21	146.76	3.12
alpha/beta-Hydrolases	53474	22	29	302.28	1.87
Cytochrome c	46626	8	21	116.14	1.6
E Set domains	81296	17	33	120.21	2.49
FAD/NAD(P)-binding	51905	5	21	244.1	1.47
Fibronectin type	49265	1	24	103.42	1.52
Homeodomain-like	46689	10	24	72.17	2.43
Immunoglobulin	48726	4	47	103.23	1.63
NAD(P)-binding	51735	10	49	202.67	1.89
Nucleic acid-binding	50249	10	44	120.86	2.05
P-loop	52540	18	70	257.64	3.99
S-adenosyl	53335	20	24	255.92	1.92
Thioredoxin-like	52833	12	29	121.72	1.88
Viral coat	49611	4	29	271.07	3.43
Winged helix	46785	35	48	92.65	2.33
